# Quadriceps Strength Loss Following Total Knee Arthroplasty as a Predictor of Three-Month Strength Recovery: A Secondary Analysis of a Randomized Controlled Trial

**DOI:** 10.7759/cureus.68244

**Published:** 2024-08-30

**Authors:** Yusuke Kubo, Daisuke Fujita, Shuhei Sugiyama, Rie Takachu, Takeshi Sugiura, Masahiro Sawada, Kohtaro Yamashita, Kaori Kobori, Makoto Kobori

**Affiliations:** 1 Department of Rehabilitation Medicine, Kobori Orthopedic Clinic, Hamamatsu, JPN; 2 Department of Physical Therapy, Fukuoka International University of Health and Welfare, Fukuoka, JPN; 3 Department of Orthopedics, Kobori Orthopedic Clinic, Hamamatsu, JPN

**Keywords:** rehabilitation, quadriceps muscle weakness, postoperative recovery, preoperative training, knee osteoarthritis, quadriceps muscle strength, total knee arthroplasty

## Abstract

Background and objectives

Patients often experience significant quadriceps muscle weakness immediately after total knee arthroplasty (TKA), which can persist and lead to reduced physical function, increased risk of falls, and reduced patient satisfaction. Immediate postoperative quadriceps weakness is commonly caused by several factors, such as preoperative quadriceps weakness related to knee osteoarthritis (OA) and TKA-induced quadriceps weakness. Although many interventions have focused on addressing knee OA-related quadriceps weakness, there may be fewer studies specifically investigating TKA-induced quadriceps weakness. This study aimed to clarify whether TKA-induced quadriceps weakness is a significant predictor of quadriceps strength at three months postoperatively, highlighting the clinical importance of preoperative interventions targeting this specific weakness.

Methods

This secondary analysis of a randomized controlled trial included patients aged 60-79 years with advanced knee OA who underwent unilateral TKA. The study used pooled data from two groups of 11 participants each: those receiving preoperative low-intensity resistance training with blood flow restriction and those performing low-intensity resistance training with slow movement and tonic force generation. Quadriceps strength was assessed using a pull-type handheld dynamometer preoperatively at six weeks and one week as well as postoperatively at four days, one month, and three months. TKA-induced quadriceps weakness was defined as a change in strength from one week preoperatively to four days postoperatively. Postoperative quadriceps strength gain, reflecting postoperative recovery, was defined as the strength change from four days to three months postoperatively. Correlation and multiple regression analyses were used to identify the predictors of postoperative quadriceps strength at three months. Statistical significance was set at p < 0.05.

Results

The analysis included 22 participants. The median preoperative quadriceps strength was 1.1 Nm/kg (IQR: 0.9-1.4) at six weeks and 1.3 Nm/kg (IQR: 1.1-1.4) at one week. Quadriceps strength significantly decreased immediately after TKA (median quadriceps strength dropped to 0.4 Nm/kg (IQR: 0.3-0.4) at four days postoperatively) and gradually improved over three months (median three-month postoperative quadriceps strength was 0.9 Nm/kg (IQR: 0.8-1.0)). TKA-induced quadriceps weakness was -72% (SD: 11%), and postoperative quadriceps strength gain was 210% (IQR: 98-324%). TKA-induced quadriceps weakness was strongly correlated with quadriceps strength at four days (r = 0.84, p < 0.01). The postoperative quadriceps strength at four days was significantly correlated with the quadriceps strength at three months (r = 0.51, p = 0.02). Regression analysis showed that one-week preoperative quadriceps strength, TKA-induced quadriceps weakness, and postoperative quadriceps strength gain significantly predicted quadriceps strength at three months (R² = 0.77, p < 0.001).

Conclusions

This study highlights TKA-induced quadriceps weakness as a key predictor of postoperative quadriceps strength at three months. Preoperative interventions targeting TKA-induced weakness may improve postoperative recovery of quadriceps strength and functional outcomes.

## Introduction

Total knee arthroplasty (TKA) is an effective treatment for advanced knee osteoarthritis (OA) that reduces pain and improves mobility [1,2]. However, patients often experience significant immediate postoperative quadriceps muscle weakness [3,4], which can persist long term [5,6]. This weakness is associated with reduced physical function, increased risk of falls, and reduced patient satisfaction [7-10]. Additionally, it can lead to compensatory movements during activities, such as walking, rising from and sitting in chairs, and climbing stairs, adding stress to the opposite lower extremity and lumbar spine [11-14]. Therefore, managing quadriceps weakness is crucial for the recovery and reduction of long-term negative effects.

The significant reduction in quadriceps strength immediately after surgery is commonly due to preoperative quadriceps weakness related to knee OA and TKA-induced factors, such as surgical trauma and tourniquet-induced ischemia-reperfusion (IR) injury [15-17]. Addressing these factors is essential for maintaining quadriceps strength immediately after surgery. Considerable research has focused on OA-induced quadriceps weakness in patients awaiting TKA [18-21]. Studies have shown that preoperative exercise interventions increase preoperative quadriceps strength and maintain enhanced strength postoperatively [19-21]. However, only a few studies have examined TKA-induced quadriceps weakness directly [22,23].

Our research has shown that preoperative exercise intervention can reduce quadriceps strength loss from before to four days after surgery [22]. However, it is unclear whether this benefit continues in the medium to long term postoperatively. Findings that preoperative quadriceps strength can predict physical function one year postoperatively may account for the emphasis on OA-related quadriceps weakness interventions [24]. Therefore, immediate postoperative reduction in quadriceps strength, particularly due to TKA, requires further investigation to determine its potential as a predictor of medium-to-long-term quadriceps strength. Confirming that TKA-induced quadriceps weakness predicts postoperative quadriceps strength at three months would underscore the importance of targeted preoperative interventions to address this specific weakness.

Following TKA, quadriceps strength typically shows a significant initial decrease immediately after surgery, followed by a linear increase over approximately two years [25]. This process can be influenced by various factors, including preoperative quadriceps strength, TKA-induced quadriceps weakness, and postoperative quadriceps strength gain (recovery rate). Individuals with high preoperative quadriceps strength who experience minimal quadriceps strength loss after TKA, resulting in higher immediate postoperative strength, and who also undergo effective postoperative recovery, are expected to maintain higher quadriceps strength postoperatively. In this study, we aimed to clarify whether TKA-induced quadriceps weakness serves as a significant predictor of quadriceps strength at three months postoperatively. Using multiple regression analysis, we tested the hypothesis that preoperative quadriceps strength, TKA-induced quadriceps weakness, and postoperative quadriceps strength gain would significantly predict quadriceps strength at three months postoperatively, while also accounting for potential confounding factors such as age and gender.

## Materials and methods

Study design

This secondary analysis of a randomized controlled trial compared the effects of four-week preoperative low-intensity resistance training with blood flow restriction (BFR) versus low-intensity resistance training with slow movement and tonic force generation (LST) on quadriceps strength before and after TKA. The details of the clinical trial have been published previously [26].

Participants

This secondary analysis used data from an assessor-blinded randomized controlled clinical study conducted at a Japanese orthopedic clinic between September 2019 and December 2021. The study included patients aged 60-79 years with advanced knee OA scheduled for unilateral TKA. Exclusion criteria included severe comorbidities (e.g., cancer, cardiovascular diseases, and uncontrolled diabetes), conditions affecting mobility or participation (e.g., significant motor dysfunction, psychiatric disorders, and severe obesity), prior knee surgeries, coagulation abnormalities, and active smoking. Additionally, patients opting for epidural anesthesia or those with plans to transfer postoperatively were excluded. Detailed inclusion and exclusion criteria are provided in the original study protocol [26]. The Seirei Christopher University Ethics Committee approved the study (approval number 19019), and all participants provided informed consent in accordance with the revised 2008 Helsinki Declaration. The trial was registered in the University Hospital Medical Information Network Clinical Trials Registry (UMIN000037981) and adhered to the CONSORT guidelines.

Randomization

This study employed blocked randomization to ensure evenly distributed participant assignment between the BFR and LST groups, categorized by sex and age (in decades), with block sizes of two, four, or six. A physiotherapist who was blinded to the study’s data collection and analysis was responsible for generating the randomized sequence. We utilized an assessor-blinded approach to mitigate outcome evaluation bias. Two physical therapists were designated as assessors and were unaware of the participants’ group allocations. Furthermore, they were not involved in the supervision of training sessions.

Surgical procedure and rehabilitation program

All participants underwent tricompartmental uncemented TKA using a low-contact-stress implant (LCS Complete; DePuy, Johnson & Johnson Co., New Brunswick, NJ, USA) via a mid-vastus approach performed by two experienced surgeons. Before wound closure, 1,000 mg of tranexamic acid was administered directly at the surgical site. A tourniquet (ATS 2000; Zimmer, Dover, OH, USA) was placed on the upper thigh and inflated to 300 mmHg.

Under the supervision of two experienced physiotherapists, the participants in the BFR and LST groups underwent preoperative and postoperative rehabilitation. Preoperatively, they attended two to three weekly sessions over four weeks, although some only managed one session per week. Each session started with thermotherapy using a 10-minute hot pack, followed by active and passive exercises to enhance knee mobility. The participants in both groups performed the same resistance exercises, including squats, forward lunges, and seated bilateral knee extensions. The BFR group used cuffs inflated to 100-120 mmHg to restrict blood flow to the lower limb scheduled for surgery during these exercises. In contrast, the LST group used cuffs inflated to 20 mmHg, simulating the conditions experienced by the BFR group without significantly restricting blood flow. In addition, all participants engaged in aerobic exercises on a cycle ergometer to maintain a heart rate of 100-120 bpm.

Inpatient rehabilitation began the day after surgery and included two to three daily sessions during a five-day hospital stay. These sessions incorporated quadriceps strengthening exercises with neuromuscular electrical stimulation and practical activities to enhance daily living skills, such as transitioning from sitting to standing and climbing stairs. Mobility training commenced on postoperative day 1 using a four-wheeled walker and progressed to T-handle canes by postoperative days 4 or 5. Post-discharge rehabilitation was continued with one or two sessions per week for three months. The first month post-discharge involved an intensive physical therapy program that included range-of-motion treatments, quadriceps-strengthening exercises enhanced by neuromuscular electrical stimulation, and gait training. During the second and third months, the preoperative LST and aerobic exercises were reintroduced and tailored to each patient’s physical state and pain tolerance. Guidance on home exercises, including hip abduction, calf raises, bridge exercises, and stair climbing, was provided, with patients advised to complete three sets of 10 repetitions daily for each exercise.

Outcomes

In the original study, participants underwent seven assessment sessions, which included baseline and follow-up evaluations. The initial baseline evaluation was conducted six weeks preoperatively, followed by a four-week intervention period. Further assessments were performed at one week and immediately preoperatively, as well as at 24 hours, four days, one month, and three months postoperatively. For this secondary analysis, only the quadriceps strength measurements from the original study were utilized. Quadriceps strength was assessed at six weeks and one week preoperatively, as well as at four days, one month, and three months postoperatively. Quadriceps strength was measured using a pull-type handheld dynamometer (Mobie; Sakai Medical Co., Ltd., Tokyo, Japan), following the protocol described in a previous study [4]. The participants were assessed while sitting with their hips at 90° and knees at 75°. To ensure stability during the test, participants gripped the sides of the couch. The measurement procedure started with two practice trials, followed by three maximum effort trials, each separated by 30-second intervals of rest. Of the three valid trials, the two highest readings were noted, and their averages were used for further analysis. Quadriceps strength was calculated as the maximum voluntary torque normalized to body mass, considering the length of the lower leg as the lever arm (Nm/kg). This method of measuring quadriceps strength has shown excellent intra- and inter-rater reliabilities [27]. TKA-induced quadriceps weakness was defined as the relative change in quadriceps strength from the value one week preoperatively to the value four days postoperatively. Postoperative quadriceps strength gain, reflecting the recovery of quadriceps strength postoperatively, was defined as the relative change in quadriceps strength values from four days to three months postoperatively. The relative rate of change was calculated using the formula: relative rate of change = (subsequent value - baseline value) / baseline value × 100. Two physical therapists who were blinded to the group assignments conducted the assessments throughout the study. The demographic and clinical characteristics of the participants were obtained from their medical records.

Statistical analysis

All statistical analyses were performed using IBM SPSS Statistics for Windows, Version 26.0 (Released 2019; IBM Corp., Armonk, NY, USA). Data from the parent trial were pooled for this analysis, as no differences in outcomes were evident between the groups over time. Normal distribution and homogeneity of variance in the data were confirmed using Shapiro-Wilk and Levene’s tests, respectively. The Friedman test was used to analyze changes in quadriceps strength over time owing to the non-normal distribution of data points at key intervals (one week preoperatively and one and three months postoperatively). Post hoc pairwise comparisons identified specific time points where significant changes in quadriceps strength were observed, with significance levels adjusted using the Bonferroni method to control for the family-wise error rate.

Pearson and Spearman correlation analyses were conducted to examine the relationship between TKA-induced quadriceps weakness and quadriceps strength before and after surgery (one week preoperatively and four days, one month, and three months postoperatively). Pearson correlation was used for normally distributed variables, while Spearman correlation was applied for non-normally distributed variables. Multiple regression analysis was conducted to identify predictors of three-month postoperative quadriceps strength. The dependent variable was three-month postoperative quadriceps strength. In contrast, the independent variables included quadriceps strength one week preoperatively, TKA-induced quadriceps weakness, and postoperative quadriceps strength gain, reflecting the recovery of quadriceps strength postoperatively. The regression model employed the enter method by simultaneously inserting all predictors. The Durbin-Watson statistic was checked to ensure there was no autocorrelation in the residuals. Regression model assumptions were confirmed by examining residuals, and residual normality was verified using the Shapiro-Wilk test. Age and sex were excluded from the independent variables because of the small sample size and lack of significant correlations with the dependent variable or other independent variables in the Pearson and Spearman correlation analyses. Specifically, the correlation coefficients (r) for age and sex were 0.03-0.19 and 0.02-0.24, respectively, with p-values of 0.41-0.89 and 0.28-0.94, respectively, indicating no significant correlation.

## Results

Among 379 patients, 22 were included in the study, whereas 357 were excluded for the following reasons: 208 did not meet the inclusion criteria, six canceled their surgeries, 45 declined to participate due to concerns about the new coronavirus infection at the time, which led them to limit their outdoor activities, and 98 were excluded for other reasons. The active control (LST group) and intervention (BFR group) groups included 11 participants each. All included participants (two men and 20 women) successfully completed the study protocol. Participant flow throughout the trial has been previously published [26]. The surgical procedure was performed uniformly across all participants without deviations. Similarly, there were no significant differences in the number or type of rehabilitation sessions provided between the LST and BFR groups, ensuring consistent delivery of interventions in both groups. Table 1 shows the demographic and clinical characteristics of the participants.

**Table 1:** Demographic and clinical characteristics of participants Variables following a normal distribution are presented as mean values with SDs, whereas categorical variables are presented as numbers with percentages. OA; osteoarthritis; TKA, total knee arthroplasty Adapted from Kubo et al. (2024) [26]

Demographic and clinical characteristics	Participants (n = 22)
Age (years), mean (SD)	74 (3)
Male, n (%)	4 (18)
Weight (kg), mean (SD)	56 (6)
BMI (kg/m^2^), mean (SD)	24 (3)
Current medical history, n (%)	
Diabetes	1 (5)
Hyperlipidemia	7 (32)
Hypertension	9 (41)
Contralateral knee, n (%)	
OA and TKA, n (%)	18 (82)
Quadriceps strength (Nm/kg), mean (SD)	1.2 (0.3)
Tourniquet time (min), mean (SD)	57 (8)

**Figure 1:**
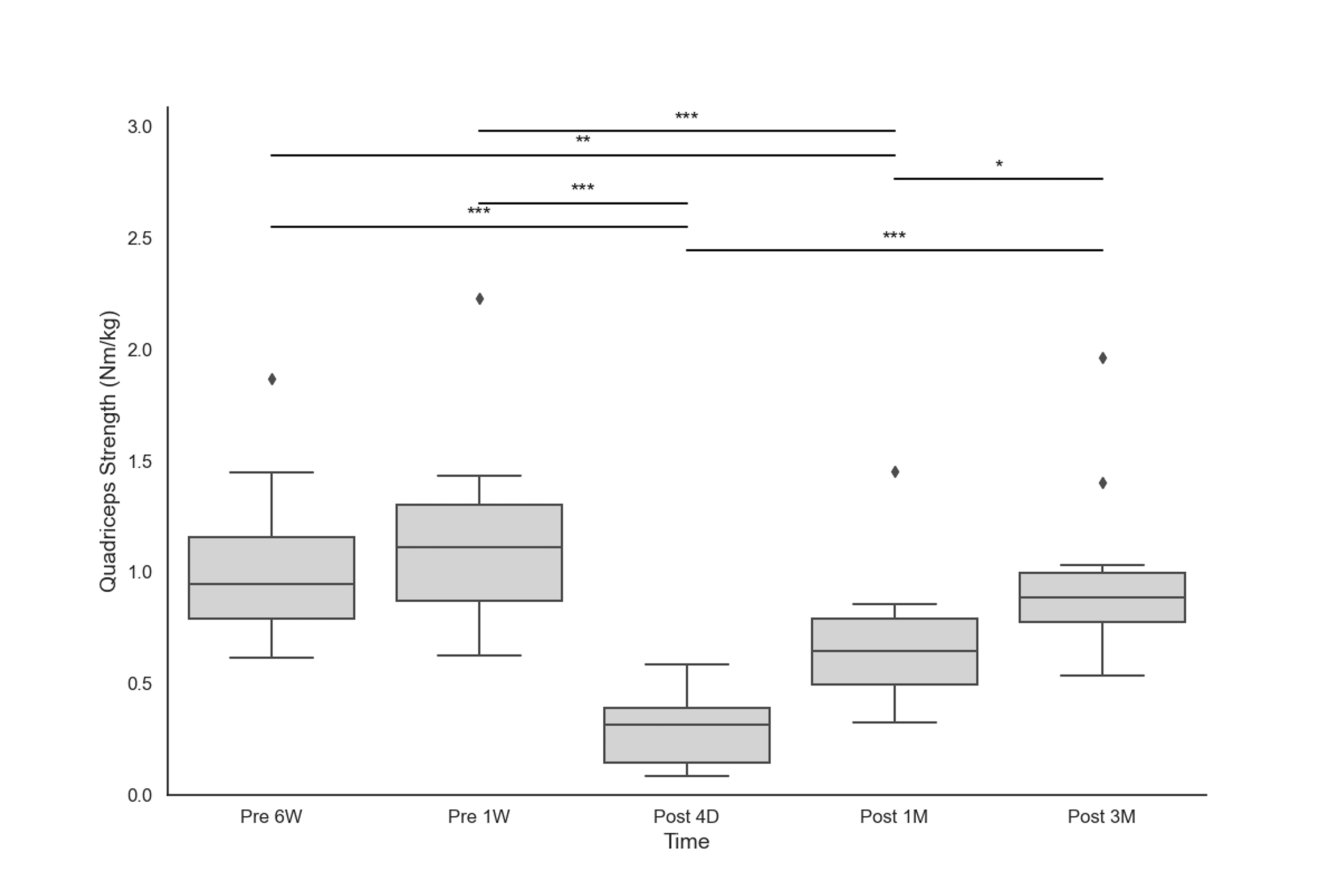
Changes in quadriceps strength before and after surgery This figure shows the changes in quadriceps strength (measured in Nm/kg) at different time points: six weeks preoperatively (Pre 6 W), one week preoperatively (Pre 1 W), four days postoperatively (Post 4D), one month postoperatively (Post 1M), and three months postoperatively (Post 3M). Box plots display the median, IQR, and outliers. Outliers were defined as data points that were more than 1.5 times the IQR above the third quartile or below the first quartile. Significant differences between the time points are indicated by asterisks (* p < 0.05, ** p < 0.01, *** p < 0.001).

Quadriceps strength data were successfully collected at each time point for all 22 participants, with no missing data for any of the measurements included in this secondary analysis. The IQRs of quadriceps strength at various time points were as follows: six weeks preoperatively, the median quadriceps strength was 1.1 Nm/kg (IQR: 0.9-1.4); one week preoperatively, the median was 1.3 Nm/kg (IQR: 1.1-1.4); four days postoperatively, the median was 0.4 Nm/kg (IQR: 0.3-0.4); one month postoperatively, the median was 0.8 Nm/kg (IQR: 0.6-0.8); and three months postoperatively, the median was 0.9 Nm/kg (IQR: 0.8-1.0). The incidence of TKA-induced quadriceps weakness was -72%, with an SD of 11%. The median postoperative quadriceps strength gain, reflecting postoperative recovery, was 210% (IQR: 98-324%). The Friedman test showed significant differences in quadriceps strength over time (p < 0.05), with post hoc pairwise comparisons using Bonferroni adjustments revealing significant differences between several time points (Figure 1).

Table 2 presents the results of correlation analyses. A significant positive correlation existed between TKA-induced quadriceps weakness and quadriceps strength at four days postoperatively (r = 0.84, p < 0.01) and between quadriceps strength at four days and three months postoperatively (r = 0.51, p < 0.05).

**Table 2:** Correlation analyses of TKA-induced quadriceps weakness and quadriceps strength measurements before and after TKA This table presents the results of Pearson and Spearman correlation analyses examining the relationship between TKA-induced quadriceps weakness (B) and quadriceps strength at various time points: one week preoperatively (A), four days postoperatively (C), one month postoperatively (D), and three months postoperatively (E). Values are presented as correlation coefficients with their corresponding p-values in parentheses. QS, quadriceps strength; TKA, total knee arthroplasty

Quadriceps strength measures	B	C	D	E
(A) QS one week preoperatively (Nm/kg)	0.06 (0.79)	0.50 (0.02)	0.70 (0.00)	0.70 (0.00)
(B) TKA-induced quadriceps strength (%)	1	0.84 (0.00)	0.42 (0.05)	0.28 (0.21)
(C) QS four days postoperatively (Nm/kg)		1	0.71 (0.00)	0.51 (0.02)
(D) QS one month postoperatively (Nm/kg)			1	0.81 (0.00)
(E) QS three months postoperatively (Nm/kg)				1

Table 3 summarizes the regression analysis results, showing the standardized beta coefficients, p-values, and variance inflation factors (VIF) for each predictor variable. The regression model predicting three-month postoperative quadriceps strength explained a significant proportion of the variance (R² = 0.77, F (3, 18) = 19.82, p < 0.001), indicating a strong overall fit. The Durbin-Watson statistic was 1.53, suggesting no significant autocorrelation in the residuals. The Shapiro-Wilk test for residual analysis showed a statistic of 0.97 (p = 0.76), confirming the normality of the residuals. Preoperative quadriceps strength (B = 0.772, 95% CI: 0.561-0.983) had the most significant impact on predicting three-month quadriceps strength, whereas TKA-induced quadriceps weakness (B = 0.014, 95% CI: 0.002-0.026) and postoperative quadriceps strength gain (B = 0.001, 95% CI: 0.000-0.001) were also important predictors. The VIF values for all variables were below five, indicating that multicollinearity was not a concern in this model.

**Table 3:** Multiple regression analysis predicting quadriceps strength at three months post-TKA This table presents the results of multiple regression analysis predicting quadriceps strength at three months post-TKA. Independent variables included preoperative quadriceps strength (Nm/kg), TKA-induced quadriceps weakness (%), and postoperative quadriceps strength gain (%). The standardized β coefficients, p-values, and VIFs are reported for each independent variable. QS, quadriceps strength; TKA, total knee arthroplasty; VIF, variance inflation factor

Independent variable	Standardized β coefficient	p-value	VIF
Preoperative quadriceps strength (Nm/kg)	0.95	p < 0.001	1.2
TKA-induced quadriceps weakness (%)	0.55	0.03	4.1
Postoperative quadriceps strength gain (%)	0.59	0.02	4.2

## Discussion

This study hypothesized that patients with high preoperative quadriceps strength, minimal quadriceps strength loss due to TKA, and effective postoperative recovery would have higher quadriceps strength postoperatively. Our findings support this hypothesis, showing that one-week preoperative quadriceps strength, TKA-induced quadriceps weakness, and postoperative quadriceps strength gain are significant predictors of three-month postoperative quadriceps strength.

In this study, quadriceps strength significantly decreased immediately after surgery and then increased linearly during the recovery period. This result is consistent with those of previous studies [19,20,25], which also reported a sharp decline in quadriceps strength immediately following TKA, followed by a gradual linear recovery. Multiple regression analysis showed that after adjusting for one-week preoperative quadriceps strength and postoperative quadriceps strength gain, TKA-induced quadriceps weakness remained a significant predictor of quadriceps strength three months postoperatively. Correlation analyses were conducted to explain the results of the multiple regression analysis as a temporal gap occurred between TKA-induced quadriceps weakness and three-month postoperative quadriceps strength. A significant positive correlation was observed between TKA-induced quadriceps weakness and quadriceps strength four days postoperatively, indicating that lower TKA-induced quadriceps weakness was associated with higher quadriceps strength four days postoperatively. Additionally, a significant positive correlation was found between quadriceps strength four days and three months postoperatively, suggesting that higher quadriceps strength four days postoperatively was associated with higher three-month postoperative quadriceps strength. By adjusting for one-week preoperative quadriceps strength and postoperative quadriceps strength gain, TKA-induced quadriceps weakness was shown to predict three-month quadriceps strength indirectly through its impact on quadriceps strength at four days postoperatively.

Additionally, in this study, due to the relatively small sample size, we conducted correlation analyses to evaluate whether age and gender, as potential confounding factors, were significantly associated with the independent variables or three-month postoperative quadriceps strength. This analysis was necessary to justify the exclusion of these variables from the final regression model. The results revealed no significant correlations between age or gender and either the independent variables or three-month postoperative quadriceps strength. Consequently, these variables were excluded from the final analysis. However, with a larger sample size, age and gender may influence overall outcomes, including recovery patterns and the relationships between key variables. Future studies with larger cohorts are needed to further investigate these factors.

The results of this study highlight the need for preoperative interventions specifically targeting TKA-induced quadriceps weakness. This weakness may be associated with surgical trauma or tourniquet-induced IR injury [16,17]. Previous studies have demonstrated that preconditioning methods, such as ischemic exposure or exercise, can mitigate IR injury [28-30]. Consequently, these findings emphasize the importance of preoperative interventions that address TKA-induced quadriceps weakness by targeting IR injuries. Although we have investigated the effects of exercise and nutritional preconditioning as preoperative interventions, research in this area remains limited [22,23,26]. In contrast, numerous studies have explored interventions aimed at increasing quadriceps strength, showing that high-intensity training improves both preoperative and postoperative quadriceps strength [19-21]. Future studies should focus on developing preoperative interventions that not only enhance quadriceps strength but also mitigate the effects of IR injury to prevent TKA-induced quadriceps weakness.

Strengths and limitations

This study makes a significant contribution to the field of postoperative recovery following TKA by addressing the underexplored yet crucial phenomenon of TKA-induced quadriceps weakness. While existing research often centers on preoperative quadriceps weakness related to knee OA, this study uniquely isolates and examines the specific impact of the surgical process itself on postoperative quadriceps weakness. By identifying key predictors of quadriceps strength at three months postoperatively - such as preoperative quadriceps strength, the extent of TKA-induced weakness, and postoperative quadriceps strength gain - this study provides critical insights into the dynamic recovery process that follows TKA. These findings offer a foundation for developing rehabilitation protocols that could potentially improve long-term outcomes by focusing on early identification and management of TKA-induced weakness.

However, this study also has several limitations. First, the relatively small sample size may have limited the generalizability and robustness of the findings. It also restricted our ability to fully account for potential confounding factors, such as age and gender, which may influence postoperative outcomes. A larger sample size in future studies is necessary to confirm these findings, better address confounding variables, and strengthen the reliability of the conclusions. Second, as this was a secondary analysis, the focus was not solely on TKA-induced quadriceps weakness, which may have introduced bias in data interpretation. The lack of control over certain aspects of the primary trial may limit the strength of the conclusions derived from this analysis. Future studies should prioritize primary data collection focused specifically on TKA-induced quadriceps weakness to reduce bias and ensure more rigorous findings. Third, a key limitation of this secondary analysis is that it relied solely on quadriceps strength as the primary outcome measure without examining its relationship to other important functional outcomes, such as gait performance, activities of daily living, and quality of life, which were measured in the parent randomized controlled trial. Although these outcomes could provide a more comprehensive understanding of postoperative recovery following TKA, we did not explore these relationships due to the absence of critical confounding factors, including contralateral limb strength, which were not assessed in the parent trial. These factors likely influence the relationship between quadriceps strength and broader functional outcomes, making it difficult to accurately adjust for these confounders in this secondary analysis. Therefore, future studies should prioritize collecting data on these additional factors and include larger sample sizes to allow for proper statistical adjustment, thereby providing a more comprehensive understanding of the recovery process following TKA. Fourth, the clinical setting constrained our ability to measure only isometric quadriceps strength, limiting our understanding of the full dynamics of muscle strength changes postoperatively. The exclusion of concentric and eccentric strength measurements may provide an incomplete picture of muscle recovery after TKA. Future research should incorporate these additional measurements to gain a more comprehensive understanding of quadriceps muscle recovery. Finally, this study only tracked quadriceps strength recovery for three months postoperatively, potentially missing the full trajectory of muscle strength recovery following TKA. Longer-term follow-up studies are essential to determine whether the short-term recovery patterns observed in this study persist, improve, or worsen over time. Future research should extend follow-up periods to assess the long-term impact of TKA-induced quadriceps weakness on sustained muscle strength and functional outcomes.

## Conclusions

This study highlights the importance of addressing TKA-induced quadriceps weakness as a significant factor in predicting quadriceps strength three months postoperatively, suggesting the need to develop preoperative interventions that specifically target TKA-induced quadriceps weakness. Given the identified limitations, further research with larger sample sizes and longer follow-up periods is needed to confirm these findings and enhance our understanding of the long-term impact of TKA-induced quadriceps weakness on quadriceps strength recovery.
